# DHA Alone, EPA + DHA, EPA + DHA + GLA, and EPA + DHA with or Without Vitamin D During Chemotherapy in Women with Breast Cancer: An Intervention- and Outcome-Stratified Systematic Review

**DOI:** 10.3390/nu18152483

**Published:** 2026-08-01

**Authors:** Kinga Fronk, Mariusz Wysokiński, Rafał Podgórski, Edyta Łuszczki

**Affiliations:** 1 Independent Researcher, ul. Wilsona 20/5, 33-100 Tarnów, Poland; fronkkinga@gmail.com; 2 Department of Fundamentals of Nursing, Chair of Nursing Development, Faculty of Health Sciences, Medical University of Lublin, 20-093 Lublin, Poland; mariusz.wysokinski@umlub.edu.pl; 3 Faculty of Medicine, University of Rzeszów, al. Tadeusza Rejtana 16C, 35-959 Rzeszow, Poland; rpodgorski@ur.edu.pl; 4 Faculty of Health Sciences and Psychology, University of Rzeszów, al. Tadeusza Rejtana 16C, 35-959 Rzeszow, Poland

**Keywords:** breast cancer, omega-3 fatty acids, eicosapentaenoic acid, docosahexaenoic acid, chemotherapy, inflammation, nutritional status, peripheral neuropathy, quality of life, GRADE

## Abstract

**Background/Objectives**: Prospective studies of omega-3 supplementation during breast cancer chemotherapy differ in intervention composition, co-supplementation, dose, treatment setting, and outcomes. We summarized and critically appraised interventions comprising DHA alone, EPA + DHA, EPA + DHA + GLA, or EPA + DHA with or without vitamin D, stratified by intervention composition and outcome domain. **Methods**: PubMed/MEDLINE, the Cochrane Library/CENTRAL, ClinicalTrials.gov, Scopus, Web of Science Core Collection, and Embase were last searched on 8 July 2026, supplemented by citation searching. Eligible studies prospectively administered EPA and/or DHA during chemotherapy and reported inflammatory/biological, nutritional/functional, quality-of-life, treatment-related, anticancer-response, or safety outcomes. Active co-supplementation was analyzed separately. Owing to clinical and methodological heterogeneity, findings were synthesized narratively; risk of bias and outcome-specific certainty were assessed using RoB 2-informed and GRADE approaches. **Results**: Fourteen reports representing ten prospective interventional trials were included. No eligible EPA-only trial was found. Interventions comprised EPA + DHA/fish oil without another active nutrient, DHA alone, EPA + DHA plus gamma-linolenic acid, and a factorial trial of EPA + DHA with or without vitamin D. One trial reported lower paclitaxel-induced neuropathy, whereas another found no benefit for paclitaxel-associated acute pain or neuropathic symptoms. Biomarker, nutritional, functional, and quality-of-life findings were inconsistent and often derived from small trials, exploratory or compliance-restricted analyses, companion reports, or co-supplementation designs. No consistent improvement in treatment response or survival was demonstrated. Serious supplementation-related adverse events were not reported, although adverse-event reporting varied. Certainty was low for safety and very low for efficacy-related domains. **Conclusions**: Current evidence is preliminary. The evaluated interventions appeared generally tolerated, but independent clinical efficacy of DHA alone, EPA + DHA without another active nutrient, EPA + DHA + GLA, or EPA + DHA with or without vitamin D during breast cancer chemotherapy remains unproven.

## 1. Introduction

Omega-3 long-chain polyunsaturated fatty acids, particularly eicosapentaenoic acid (EPA) and docosahexaenoic acid (DHA), participate in membrane organization, cellular signaling, and the regulation of inflammatory and immune responses [1,2]. These mechanisms are relevant during cancer treatment because inflammation, nutritional deterioration, and treatment-related symptoms may influence chemotherapy tolerance and patient-centered outcomes [2,3,4].

Prospective studies in women receiving chemotherapy for breast cancer have evaluated heterogeneous interventions. Some trials used mixed EPA + DHA preparations or fish oil, some used DHA alone, and others combined EPA + DHA with active nutrients such as vitamin D or gamma-linolenic acid (GLA). Treating these interventions as a single exposure can obscure biologically and clinically important differences and can lead to over-attribution of effects to EPA or DHA.

Previous reviews have considered omega-3 fatty acids in breast cancer more broadly [1], but evidence specifically arising from prospective supplementation during active chemotherapy remains fragmented. The present systematic review therefore aimed to summarize and critically appraise prospective interventional evidence for EPA alone, DHA alone, EPA + DHA or fish oil without another active nutrient, EPA + DHA + GLA, and EPA + DHA with or without vitamin D during chemotherapy in women with breast cancer. Findings were stratified by intervention composition, co-supplementation, study design, treatment setting, and outcome domain. The review question was: In adult women receiving active chemotherapy for breast cancer, what are the effects and safety of these composition-specific interventions, compared with placebo, no supplementation, standard care, or factorial comparator arms, on inflammatory/biological, nutritional/functional, patient-reported, treatment-related, anticancer-response, survival, and safety outcomes?

## 2. Materials and Methods

### 2.1. Design, Reporting Framework, and Protocol Status

This systematic review was prepared in accordance with the PRISMA 2020 statement [5]. The review was not prospectively registered, and no separate protocol was prepared or published. Eligibility criteria were defined according to the PICO framework before the final eligibility assessment. Intervention-composition categories and the outcome-specific GRADE assessment were added post hoc to strengthen the final synthesis; these additions did not alter the eligibility criteria.

### 2.2. Eligibility Criteria

The population comprised adult women (aged 18 years or older) with breast cancer receiving active systemic chemotherapy in the neoadjuvant, adjuvant, or metastatic setting. Eligible interventions prospectively administered one of the following during chemotherapy: EPA alone; DHA alone; a defined EPA + DHA or fish-oil formulation without another active nutrient; EPA + DHA + GLA; or EPA + DHA with or without vitamin D in a factorial design. Eligibility was not restricted by dose, formulation, or duration. Studies that combined EPA + DHA with another active nutrient were eligible only when EPA + DHA was a prespecified intervention component; these studies were analyzed separately and were not used to infer the independent effect of EPA or DHA.

Eligible comparators included placebo, no supplementation, standard care, or another arm in a randomized factorial design. Randomized controlled trials, controlled prospective interventional studies, and prospective single-arm phase II studies were eligible. The single-arm study was retained as exploratory, non-comparative evidence because it prospectively evaluated DHA during active chemotherapy and reported clinically relevant outcomes.

Outcomes of interest included inflammatory, immune, and biological markers; nutritional status, body weight, body composition, and functional outcomes; quality of life and other patient-reported outcomes; chemotherapy-induced peripheral neuropathy (CIPN), paclitaxel-associated acute pain syndrome (P-APS), and other treatment-related symptoms or toxicities; anticancer treatment response and survival; and safety or tolerability. Reviews, meta-analyses, protocols without outcome data, registry records without results, preclinical or in vitro studies, observational studies without an assigned supplementation intervention, case reports, or case series, and publications without extractable clinical outcome data were excluded. Studies in which supplementation was administered only before chemotherapy or only after completion of treatment were also excluded.

### 2.3. Information Sources and Search Strategy

All database and register searches were updated and completed on 8 July 2026. PubMed/MEDLINE, the Cochrane Library/CENTRAL, ClinicalTrials.gov, Scopus, Web of Science Core Collection, and Embase were searched. The PubMed/MEDLINE, CENTRAL, and ClinicalTrials.gov strategies were retained as source-specific searches, whereas broader Boolean strategies were used in Scopus, Web of Science, and Embase. The exact source-specific strings, interfaces, limits, final search dates, and record counts are reported in Supplementary File S2.

Backward and forward citation searching was performed for included reports and relevant reviews, and trial registrations and companion publications were cross-checked. A final trial-identifier/citation audit identified one additional unique eligible report: the full peer-reviewed DHALYA report by Mantha et al. (NCT01548534), which was not counted among the 673 database/register records.

### 2.4. Record Management, Study Selection, and Retrieval

Database and register exports were combined, and duplicates were identified using DOI and normalized-title matching followed by manual verification. Titles and abstracts were screened by K.F.; full-text eligibility decisions were made by K.F. and checked by E.Ł. Uncertainties were resolved by discussion. No automation or machine-learning tools were used for study selection.

The database and register searches identified 673 records. After removal of 166 duplicates, 507 records were screened, and 486 were excluded at title/abstract screening. Twenty-one reports were sought and retrieved in full, and all 21 were assessed for eligibility. Eight reports were excluded: two because the population was mixed and breast cancer data were not separately extractable, two because the design was ineligible or did not evaluate an assigned supplementation effect, two because they were protocol or registry records without outcome data, and two because they were conference abstracts or duplicate preliminary reports. Thirteen reports were included from the database/register searches. One additional full peer-reviewed report was identified through trial-identifier/citation searching and was included. In total, 14 reports representing 10 unique trial clusters were included. The full retrieval and exclusion log is provided in Supplementary File S2.

Multiple reports originating from the same clinical trial were grouped under one trial identifier and were not double counted. The two Almassri reports were treated as companion reports from one factorial trial, and the four DHA-WIN reports were treated as companion reports from one randomized trial.

### 2.5. Data Collection and Data Items

K.F. extracted data using a standardized form, and consistency was verified by M.W. and E.Ł. Extracted variables included country, study design, eligibility criteria, treatment setting, chemotherapy regimen, numbers enrolled or randomized and analyzed or completed, intervention composition, separate EPA and DHA doses when available, co-supplementation, duration, comparator, outcome definitions and time points, main numerical results, adverse events, trial registration, funding, and conflicts of interest. Trial-level characteristics were recorded once, whereas report-specific outcomes from companion publications were retained separately.

### 2.6. Risk-of-Bias Assessment

Randomized trials were assessed using an outcome-specific, structured domain-based approach informed by Cochrane RoB 2 [6], considering the randomization process, deviations from intended interventions, missing outcome data, outcome measurement, and selection of the reported result. Judgments were made for the principal result(s) contributing to each synthesis and used only the standard categories low risk, some concerns, or high risk. Separate result-level rows were used when a trial contributed outcome sets with materially different bias considerations. The prospective single-arm phase II study was assessed separately for prospective design, completeness of follow-up, outcome ascertainment, and suitability for causal or comparative inference. K.F. performed the assessments, and E.Ł. checked them; disagreements were resolved by discussion. Detailed judgments and rationales are reported in Supplementary File S2.

### 2.7. Synthesis Methods

Findings were synthesized narratively. Interventions were grouped as: (1) EPA + DHA or fish oil without another active nutrient; (2) DHA alone; (3) EPA + DHA plus GLA; and (4) EPA + DHA with or without vitamin D in a factorial design. Within each intervention category, results were organized by outcome domain.

Meta-analysis was not undertaken because studies differed substantially in intervention composition, dose, duration, chemotherapy setting, comparator, outcome definitions, reporting metrics, and assessment time points. Most outcome domains were informed by one or two trial clusters, and several outcomes were reported only in companion publications from the same trial. For dichotomous outcomes, event counts, percentages, odds ratios, hazard ratios, or other reported estimates were extracted; for continuous outcomes, between-group differences, adjusted changes, confidence intervals, or *p*-values were extracted where available. Statistical heterogeneity, subgroup analysis, meta-regression, and sensitivity analysis were therefore not performed. Formal assessment of small-study or publication bias was not considered informative because there were only ten trial clusters and outcome reporting was highly heterogeneous.

### 2.8. Certainty of Evidence

**Figure 1:**
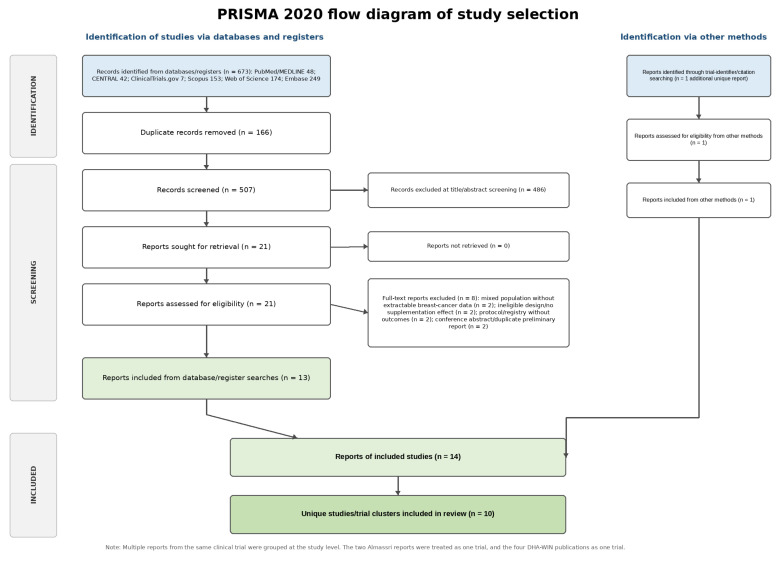
PRISMA 2020 flow diagram of study selection. Multiple reports from the same clinical trial were grouped at the study level.

Certainty was assessed by outcome domain using GRADE principles [7], considering risk of bias, inconsistency, indirectness, imprecision, and possible missing results. Because quantitative pooling was not performed, judgments were based on the consistency, directness, and precision of the available narrative evidence (Figure 1). K.F. performed the initial GRADE assessments, which were checked by M.W. and E.Ł.; disagreements were resolved by discussion. Detailed justifications are provided in Supplementary File S3.

## 3. Results

### 3.1. Study and Report Structure

The 14 included reports were published between 2009 and 2025 and represented ten prospective interventional trial clusters [8,9,10,11,12,13,14,15,16,17,18,19,20,21]. Nine trial clusters were randomized studies: seven placebo- or matched-control trials, one no-supplementation controlled trial, and one open-label factorial trial. One study was a prospective open-label single-arm phase II trial. Participant denominators varied across reports; Table 1 therefore distinguishes the number enrolled or randomized from the number analyzed or completing the relevant assessment rather than presenting one potentially misleading aggregate participant total.

Two trial clusters generated companion reports. The Almassri trial contributed a report on nutritional outcomes and a companion report on quality of life and inflammatory markers [13,19]. DHA-WIN contributed a primary trial report and three companion reports addressing cytokines/oxylipins/tumor-infiltrating lymphocytes, peripheral blood immune cells, and quality of life/exercise behavior [14,15,16,17].

### 3.2. Intervention Categories

Six trial clusters evaluated EPA + DHA or fish oil without another active nutrient [8,9,10,18,20,21]. Two trial clusters evaluated DHA alone: the non-comparative Bougnoux phase II study and the placebo-controlled DHA-WIN trial [11,14,15,16,17]. One trial evaluated EPA + DHA together with GLA [12], and one factorial trial evaluated EPA + DHA with or without vitamin D [13,19]. No eligible EPA-only intervention was identified. This composition-based grouping was applied consistently to avoid combining biologically distinct exposures and to prevent co-supplementation trials from being interpreted as evidence of the independent efficacy of EPA, DHA, or EPA + DHA.

### 3.3. Outcome-Specific Narrative Synthesis

#### 3.3.1. CIPN, Paclitaxel-Associated Pain, and Other Treatment-Related Outcomes

Two placebo-controlled trials evaluated paclitaxel-related neuropathy or acute pain [8,9]. Ghoreishi et al. reported a lower incidence of CIPN among 57 analyzed participants: CIPN occurred in 9/30 participants receiving the DHA-predominant EPA + DHA formulation and in 16/27 receiving placebo (30.0% vs. 59.3%; OR for CIPN incidence 0.30, 95% CI 0.10–0.88; *p* = 0.029; author-reported NNT = 3) [8]. However, 12 of 69 randomized participants were not included in the analysis, and the between-group difference in neuropathy severity did not reach conventional statistical significance (*p* = 0.054).

Tawfik et al. did not confirm a protective effect. P-APS occurred in 68.0% versus 62.5% during the first treatment week (*p* = 0.77) and in 84.0% versus 87.5% over 12 weeks (*p* = 1.0); the CIPN score was numerically higher in the EPA + DHA ethyl-ester group (12.8 vs. 8.4; *p* = 0.21) [9]. Therefore, the neuropathy and acute pain evidence was inconsistent and imprecise.

De la Rosa Oliva et al. found no meaningful between-group difference for most chemotherapy-related toxicity or symptom outcomes, although xerostomia improved with EPA + DHA (*p* = 0.032) [18]. Suzumura et al. reported favorable respiratory muscle, 6 min walk, lactate, and lipid outcomes after 60 days of fish oil, but the study was small and used a no-supplementation rather than placebo control [20].

#### 3.3.2. Inflammatory, Immune, and Biological Markers

Darwito et al. reported greater reductions in Ki-67 and VEGF expression after neoadjuvant CAF plus fish-oil omega-3 than after CAF plus placebo (*p* = 0.032 and *p* = 0.041) [10]. The clinical meaning of these biomarker differences is uncertain because the trial was small and reported multiple biomarker and survival outcomes.

In the EPA + DHA + GLA trial, end-of-study IL-6 was lower in the intervention group after adjustment for baseline values (*p* < 0.01), whereas IL-8, IL-10, and TNF-alpha did not differ between groups [12]. Because GLA was co-administered, the IL-6 finding cannot be attributed to EPA or DHA alone.

DHA-WIN companion reports described changes in cytokines, oxylipins, tumor-infiltrating lymphocytes, and peripheral immune-cell phenotypes [15,16]. For example, the neutrophil-to-lymphocyte ratio was maintained in the DHA group but increased in the placebo group (interaction *p* = 0.02) [16]. These analyses were mechanistic, involved multiple biomarkers, and did not establish a corresponding clinical benefit. The Almassri companion report also described inflammatory-marker improvements in an open-label factorial trial of EPA + DHA with or without vitamin D; the independent contribution of EPA + DHA was therefore uncertain [19].

Mantha et al. reported the full peer-reviewed DHALYA trial (NCT01548534), a randomized, double-blind, matched-control study in women with metastatic HER2-negative, hormone-receptor-positive breast cancer [21]. Fish-oil supplementation rapidly increased plasma EPA and DHA, and compliance-restricted analyses suggested a change in protein metabolism within approximately 10 days, whereas proinflammatory cytokines decreased only after longer supplementation. These mechanistic findings require caution because the trial was terminated early, only 46 of 63 randomized participants completed the 3-month assessment, compliance declined over time, and the study was underpowered for its planned survival endpoint.

#### 3.3.3. Nutritional Status, Body Composition, and Functional Outcomes

The clearest nutritional signal came from the combined EPA + DHA + vitamin D arm of the Almassri factorial trial [13]. After nine weeks, the combined group had a lower PG-SGA score than the control group (*p* < 0.001) and a higher BMI (*p* = 0.032). Serum albumin increased within the combined group (*p* = 0.042), but the between-group comparison was not significant. Because the trial was open-label and included vitamin D as an active co-intervention, these findings cannot establish an independent EPA + DHA effect.

De la Rosa Oliva et al. found no significant benefit for most body-composition or cardiometabolic outcomes [18]. Arsic et al. found no meaningful changes in body composition in either group [12]. Suzumura et al. reported improvements in respiratory muscle strength/endurance and 6 min walk performance, but this outcome domain was represented by a single small no-placebo study [20].

#### 3.3.4. Quality of Life and Patient-Reported Outcomes

Quality-of-life findings were mixed. The Almassri companion report described improvements in selected quality-of-life outcomes across the EPA + DHA-only, vitamin-D-only, and combined EPA + DHA + vitamin D arms [19], but the open-label factorial design permits expectation effects and does not isolate the effect of EPA + DHA. In DHA-WIN, there was no significant adjusted between-group change in any reported QoL indicator, and DHA did not affect changes in aerobic exercise volume or resistance-training frequency [17]. The de la Rosa Oliva trial was also largely neutral for patient-reported symptoms apart from xerostomia [18].

#### 3.3.5. Anticancer Treatment Response and Survival

Bougnoux et al. reported an objective response rate of 44%, median time to progression of 6 months, and median overall survival of 22 months in 25 women receiving DHA with FEC [11]. Because there was no concurrent comparator, these findings are hypothesis-generating and cannot establish treatment enhancement.

Darwito et al. reported longer overall survival (HR 0.411, 95% CI 0.201–0.840) and disease-free survival (HR 0.439, 95% CI 0.222–0.869) [10]. These statistically significant results should be interpreted cautiously because they arose from a single-center trial of 48 participants, with multiple outcomes and limited methodological reporting. The primary DHA-WIN report did not establish a consistent improvement in pathological response, proliferation, or longer-term clinical outcomes [14]. The DHALYA trial was terminated early and was too small to evaluate its planned progression-free survival endpoint [21]. Taken together, the evidence does not support a reliable anticancer-efficacy claim (Table 2 and Table 3).

#### 3.3.6. Safety and Tolerability

Across the distinct intervention categories (DHA alone, EPA + DHA/fish oil without another active nutrient, EPA + DHA + GLA, and EPA + DHA with or without vitamin D), supplementation was generally described as tolerated within the studied doses and settings, and no serious adverse event was directly attributed to supplementation. Bougnoux et al. reported no DHA-related adverse events, although GRADE 3/4 neutropenia occurred in 80% and was attributed to the chemotherapy context [11]. In the Almassri trial, four mild participant-reported adverse events occurred among 88 analyzed participants, mainly nausea and abdominal discomfort in the EPA + DHA-only and combined EPA + DHA + vitamin D groups [13]. Safety certainty remained low because adverse-event definitions and reporting completeness varied and most studies were small.

### 3.4. Risk of Bias and Certainty of Evidence

Risk of bias was heterogeneous across the assessed results. High-risk features included attrition with per-protocol analysis, open-label no-placebo comparisons for subjective outcomes, substantial missing outcome data, absence of a concurrent comparator, and selective emphasis on multiple exploratory outcomes. Outcome-specific domain judgments, using only the categories low risk, some concerns, and high risk, are provided in Supplementary File S2.

## 4. Discussion

### 4.1. Principal Findings

This intervention- and outcome-stratified review found that prospective evidence across DHA alone, EPA + DHA/fish oil without another active nutrient, EPA + DHA + GLA, and EPA + DHA with or without vitamin D during breast cancer chemotherapy remains small, heterogeneous, and uncertain. GRADE certainty was low for safety and very low for every efficacy-related domain. The most consistent observation was the absence of a clear serious supplementation-related safety signal within the studied doses; however, incomplete adverse-event reporting and small samples preclude a firm safety conclusion. Evidence for CIPN, inflammatory biomarkers, nutritional status, functional outcomes, quality of life, and anticancer efficacy was inconsistent or derived from small, indirect, or exploratory studies and was therefore judged very low certainty. The newly included DHALYA full report added a compliance-dependent mechanistic signal but did not provide adequately powered survival evidence.

Separating intervention composition materially changed interpretation. The EPA + DHA + GLA trial cannot isolate the effect of EPA + DHA, and the strongest nutritional findings arose from a combined EPA + DHA + vitamin D arm. Similarly, the DHA-only evidence included one single-arm metastatic study and one randomized neoadjuvant trial with substantial attrition and multiple companion analyses. No eligible EPA-only trial was identified.

### 4.2. Interpretation by Intervention Type

Among EPA + DHA or fish-oil trials without another active nutrient, neuropathy results conflicted: Ghoreishi et al. reported a lower CIPN incidence, whereas Tawfik et al. found no benefit for P-APS or CIPN [8,9]. This yielded very-low-certainty evidence for CIPN/P-APS because of inconsistency, attrition, and imprecision. The de la Rosa Oliva trial was neutral for most toxicity and body-composition outcomes, with improvement confined to xerostomia [18]. Suzumura et al. added a functional signal, but the functional/respiratory evidence was graded very low certainty because it rested on a single small no-placebo study [20]. Mantha et al. added a rapid, compliance-dependent protein-metabolism signal followed by later cytokine changes, but early termination and attrition substantially limited inference; certainty for the inflammatory/biological domain remained very low [21].

DHA-only evidence was also insufficient for clinical recommendations. Bougnoux et al. generated a response and survival hypothesis but lacked a comparator [11]. DHA-WIN contributed valuable feasibility, mechanistic, and safety information, but its clinical outcome data did not demonstrate a consistent treatment benefit [14,15,16,17]. Accordingly, certainty for anticancer response and survival was very low. Co-supplementation studies generated biologically plausible signals, yet their findings should be attributed to the complete intervention rather than to EPA or DHA alone [12,13,19]; certainty for the inflammatory/biological, nutritional/body-composition, and quality-of-life domains was also very low.

### 4.3. Relation to Previous Literature

The conservative conclusions are compatible with broader oncology-nutrition reviews, which suggest that omega-3 fatty acids may influence inflammatory and body-weight-related outcomes in selected cancer populations but emphasize heterogeneity in dose, duration, disease stage, and co-interventions [22,23,24]. The present review adds a narrower chemotherapy-specific perspective and shows that intervention composition and trial design are central to interpretation. Evidence from other cancer populations or cachexia settings should not be directly extrapolated to women receiving chemotherapy for breast cancer.

### 4.4. Strengths and Limitations

Strengths include expansion of the search to six databases/registers, full retrieval reassessment, explicit grouping of companion reports, separation of DHA-only, EPA + DHA, and co-supplementation strategies, reporting of randomized and analyzed denominators separately, and outcome-specific certainty assessment.

Important review-process limitations remain. The review was not prospectively registered, and no protocol was prepared. One reviewer performed primary screening and extraction, with senior checking rather than fully independent duplicate procedures. Although all database and register searches were updated to a common final date of 8 July 2026, the PubMed/MEDLINE, CENTRAL, and ClinicalTrials.gov strategies were narrower than the broad Boolean strategies used in Scopus, Web of Science, and Embase. Intervention stratification and GRADE were introduced post hoc. Although all 21 reports from database/register screening and the one additional report identified through trial-identifier/citation searching were retrieved and assessed, selective publication and missing results cannot be excluded.

The evidence base itself was limited by small samples, heterogeneous chemotherapy regimens and doses, variable follow-up, active co-supplementation, open-label or non-comparative designs, substantial attrition in some trials, multiple companion publications, and inconsistent outcome definitions and reporting. These limitations prevented meaningful meta-analysis and substantially lowered certainty.

### 4.5. Implications for Research and Practice

For clinical practice, given low certainty for safety and very low certainty for every efficacy-related domain, the available data support only a cautious statement that the studied interventions were generally tolerated. No eligible EPA-only trial was identified. For the evaluated categories, the data do not justify recommending DHA alone, EPA + DHA/fish oil, EPA + DHA + GLA, or EPA + DHA with or without vitamin D to prevent CIPN, improve quality of life or nutritional status, or enhance anticancer efficacy during breast cancer chemotherapy.

Future research should prioritize adequately powered, prospectively registered multicenter RCTs. Trials should test standardized, analytically verified EPA-only and DHA-only formulations in separate arms and evaluate standardized, analytically verified EPA + DHA formulations in distinct arms; EPA + DHA + GLA and EPA + DHA with or without vitamin D should be analyzed separately. Protocols should prespecify and separately report EPA and DHA doses, the EPA:DHA ratio, formulation and source, purity/oxidation and stability characteristics, placebo composition, exposure or adherence biomarkers, chemotherapy regimen, and standardized clinically meaningful outcomes. Intention-to-treat analyses, prespecified adverse-event definitions, complete reporting of randomized and analyzed denominators, and harmonized assessment time points are needed. Factorial designs are especially appropriate when an active nutrient such as vitamin D is co-administered.

## 5. Conclusions

Prospective interventional evidence across DHA alone, EPA + DHA/fish oil without another active nutrient, EPA + DHA + GLA, and EPA + DHA with or without vitamin D during chemotherapy in women with breast cancer remains preliminary. GRADE certainty was low for safety and very low for all efficacy-related outcomes. The evaluated interventions appeared generally tolerated within the studied doses and settings, but the evidence is insufficient to establish independent clinical efficacy for inflammatory, nutritional, functional, quality-of-life, neuropathy, toxicity, treatment-response, or survival outcomes. Adequately powered multicenter RCTs that test standardized EPA-only and DHA-only formulations in separate arms, evaluate clearly characterized EPA + DHA formulations, and apply harmonized outcome reporting are required.

## Figures and Tables

**Table 1:** Characteristics and principal findings of included trial clusters.

Study/Reports	Design and Sample	Intervention and Comparator	Principal Results	Key Limitations/Safety
**S1; Ghoreishi et al. [8]**	Iran; double-blind placebo-controlled RCT; 69 randomized (35/34); 57 analyzed (30/27); breast cancer during paclitaxel.	DHA-predominant EPA + DHA product, 640 mg three times daily (54% DHA, 10% EPA; article-reported total omega-3 1244.1 mg/day) during paclitaxel and for 1 month after; placebo.	CIPN occurred in 9/30 (30.0%) vs. 16/27 (59.3%); OR for CIPN incidence 0.30 (95% CI 0.10–0.88), *p* = 0.029; severity difference *p* = 0.054; author-reported NNT = 3.	NCT01049295. Attrition and per-protocol analysis increased risk of bias.
**S2; Bougnoux et al. [11]**	France; prospective open-label single-arm phase II; 25 enrolled/analyzed; rapidly progressing visceral metastatic disease; first-line FEC.	DHA 1.8 g/day, starting 7–10 days before chemotherapy and continuing for approximately 5 months; no concurrent comparator.	Objective response 44% (95% CI 24.5–63.5); median time to progression 6 months; median overall survival 22 months.	No DHA-related adverse events; GRADE 3/4 neutropenia 80%. Non-comparative evidence only.
**S3; Darwito et al. [10]**	Indonesia; double-blind RCT; 48 randomized (24/24); stage IIIB disease; three cycles of neoadjuvant CAF followed by mastectomy.	Fish-oil omega-3 1 g/day for 51 days vs. placebo.	Reported greater Ki-67 and VEGF reductions (*p* = 0.032 and *p* = 0.041); OS HR 0.411 (95% CI 0.201–0.840); DFS HR 0.439 (95% CI 0.222–0.869).	Small trial with multiple outcomes and limited reporting; survival findings require external confirmation.
**S4; Tawfik et al. [9]**	United States; pilot double-blind placebo-controlled RCT; 60 randomized; 49 evaluable; weekly paclitaxel.	EPA + DHA ethyl esters (Lovaza) 4 g/day from 1 week before paclitaxel until treatment stopped; placebo.	P-APS in week 1: 68.0% vs. 62.5%, *p* = 0.77; over 12 weeks: 84.0% vs. 87.5%, *p* = 1.0; CIPN score 12.8 vs. 8.4, *p* = 0.21.	NCT01821833. Pilot was underpowered and did not support efficacy.
**S5; Arsic et al. [12]**	Serbia; double-blind placebo-controlled RCT; 32 randomized (16/16); 29 completed; postmenopausal stage IIA-IIIA disease during adjuvant anthracycline-based chemotherapy.	EPA 400 mg + DHA 600 mg + GLA 351 mg/day for 12 weeks; mineral-oil placebo.	Adjusted end-of-study IL-6 was lower in the intervention group (*p* < 0.01); no between-group differences for IL-8, IL-10, or TNF-alpha; body composition was unchanged.	NCT03516253. Effect cannot be attributed to EPA + DHA independently of GLA.
**S6; Almassri et al. [13,19]**	Palestine; open-label 1:1:1:1 randomized factorial trial; 96 recruited/randomized; 88 analyzed (22/arm); stage II-III disease receiving AC chemotherapy.	Four groups: EPA 360 mg + DHA 240 mg/day; vitamin D3 50,000 IU/week; combined EPA + DHA + vitamin D; or no supplementation; 9 weeks.	Combined group: lower PG-SGA score vs. control (*p* < 0.001), higher BMI vs. control (*p* = 0.032); albumin improved within group (*p* = 0.042) but not between groups. Companion report described QoL/inflammatory signals.	NCT05331807. Four mild AEs among 88 participants; open-label/co-supplementation limit attribution.
**S7; DHA-WIN [14,15,16,17]**	Canada; phase II double-blind placebo-controlled RCT; 76 randomized; 49 completed; non-metastatic breast cancer during six cycles of neoadjuvant chemotherapy.	Algae-derived DHA 4.4 g/day for 18 weeks vs. corn/soy-oil placebo.	No significant adjusted between-group change in QoL; exercise interactions *p* = 0.53 and *p* = 0.15. Mechanistic reports described immune/cytokine signals, but the primary trial did not establish clinical anticancer benefit.	NCT03831178. Adherence 86% vs. 78%; high attrition increased uncertainty.
**S8; de la Rosa Oliva et al. [18]**	Mexico; double-blind placebo-controlled RCT; 53 randomized; 52 analyzed; locally advanced disease; AC followed by paclitaxel with or without trastuzumab.	EPA 1.6 g + DHA 0.8 g/day for 6 months vs. placebo.	No significant between-group differences in most hematologic toxicity, body-composition, cardiometabolic, or symptom outcomes; xerostomia improved (*p* = 0.032).	No clear safety signal attributable to supplementation; multiple outcomes and small sample.
**S9; Suzumura et al. [20]**	Brazil; randomized controlled study; 32 randomized/analyzed (15 control; 17 fish oil); women aged 33–62 years at the beginning of chemotherapy.	Fish oil 4 g/day (approximately EPA 0.8 g + DHA 0.4 g/day) for 60 days vs. no supplementation.	Improved maximal inspiratory/expiratory pressure, maximum voluntary ventilation, 6 min walk performance, lactate, and selected lipid measures (reported *p* ≤ 0.05).	No placebo and small sample; adverse events and trial registration were not reported in the full text; functional findings have not been replicated.
**S10; Mantha et al. (DHALYA) [21]**	France; randomized double-blind matched-control phase III trial, terminated early; 63 randomized (32 control/31 fish oil); 46 completed; metastatic HER2-negative, HR-positive disease; planned first-line chemotherapy.	Isocaloric oral nutritional supplement, three 200 mL cans/day (EPA 2.64 g + DHA 1.56 g/day), starting approximately 10 days before chemotherapy and continuing for 3 months; matched control supplement containing copra oil.	Plasma EPA/DHA increased rapidly; compliance-restricted analyses suggested an early protein-metabolism change, whereas proinflammatory cytokines decreased only after longer supplementation.	NCT01548534. Trial terminated early; 46/63 completed; declining compliance, exploratory mechanistic analyses, and underpowered survival endpoint.

Abbreviations: AC, doxorubicin/cyclophosphamide; CAF, cyclophosphamide/doxorubicin/5-fluorouracil; CI, confidence interval; CIPN, chemotherapy-induced peripheral neuropathy; DFS, disease-free survival; DHA, docosahexaenoic acid; EPA, eicosapentaenoic acid; FEC, 5-fluorouracil/epirubicin/cyclophosphamide; GLA, gamma-linolenic acid; HER2, human epidermal growth factor receptor 2; HR, hormone receptor; NNT, number needed to treat; OS, overall survival; P-APS, paclitaxel-associated acute pain syndrome; PG-SGA, Patient-Generated Subjective Global Assessment; QoL, quality of life; RCT, randomized controlled trial.

**Table 2:** Outcome-by-intervention evidence map. Entries summarize direction and limitations of evidence and do not imply confirmed efficacy.

Intervention Category	Inflammatory/ Biological	CIPN/ Treatment Symptoms	Nutritional/ Functional	QoL/PROs	Response/ Survival	Safety
EPA + DHA/fish oil without active co-supplement	Mixed biomarker and mechanistic signals, including a compliance-restricted protein-metabolism/cytokine signal; no confirmed clinical translation	Mixed: one favorable CIPN trial, one neutral P-APS/CIPN trial; xerostomia signal	Mostly neutral body composition; one unreplicated functional/respiratory study	Limited or neutral where measured	No consistent efficacy; one small trial reported survival signals, and DHALYA was underpowered for its planned survival endpoint	Generally tolerated; reporting variable
DHA alone	Exploratory immune/cytokine changes in DHA-WIN	Not a primary evidence base for CIPN	Limited	Neutral for DHA-WIN QoL/exercise	Single-arm response data and negative/uncertain randomized clinical efficacy	Generally tolerated
EPA + DHA + GLA	IL-6 reduction; the effect of EPA + DHA cannot be isolated from GLA	Not assessed	No clear body-composition effect	Not assessed	Not assessed	Generally tolerated
EPA + DHA with or without vitamin D (factorial trial)	Inflammatory-marker signals; attribution to EPA + DHA limited	Not assessed	Combined EPA + DHA + vitamin D arm improved PG-SGA/BMI; indirect	Selected QoL signals across factorial arms; very indirect	Not assessed	Mild GI events reported

**Table 3:** Summary of outcome-specific certainty of evidence. Full GRADE judgments are provided in Supplementary File S3.

Outcome Domain	Contributing Evidence	Narrative Effect Summary	Certainty	Main Reasons
Safety/tolerability	Ten trial clusters; adverse-event reporting varied	No serious supplementation-related adverse event was reported, but definitions and completeness differed.	Low	Serious risk of bias and imprecision.
Inflammatory/immune/biological markers	Darwito; Arsic; DHA-WIN companion reports; Almassri companion report; Mantha/DHALYA	Several biomarkers and mechanistic signals, but results were exploratory, heterogeneous, sometimes compliance-restricted, and sometimes co-supplemented.	Very low	Risk of bias, inconsistency, indirectness, and imprecision.
CIPN/P-APS	Ghoreishi and Tawfik; 106 analyzed participants	One favorable trial and one neutral pilot trial.	Very low	Inconsistency, attrition, and imprecision.
Nutritional status/body composition	Almassri, de la Rosa Oliva, and Arsic; 169 analyzed/completed participants	Positive nutritional-status signal was confined mainly to the combined EPA + DHA + vitamin D arm; placebo-controlled body-composition findings in de la Rosa Oliva and Arsic were largely neutral.	Very low	Risk of bias, indirectness, inconsistency, and imprecision.
Functional/respiratory/lipid-lactate	Suzumura; 32 participants	Favorable results in a single no-placebo study.	Very low	Single small study, performance bias, and severe imprecision.
Quality of life/PROs	Almassri companion; DHA-WIN/Douglas; de la Rosa Oliva	Mixed findings; strongest positive signal arose from open-label co-supplementation.	Very low	Risk of bias, indirectness, inconsistency, and imprecision.
Anticancer response/survival	Bougnoux; Darwito; DHA-WIN; Mantha/DHALYA	No consistent hard clinical benefit; favorable results came from non-comparative or small single-center evidence, and DHALYA was underpowered for survival.	Very low	Very serious risk of bias, inconsistency, and imprecision.

## Data Availability

All data used in this systematic review were derived from published reports cited in the manuscript. The complete search strategies, full-text decisions, extracted study-level data, risk-of-bias judgments, and GRADE assessments are provided in Supplementary Files S1–S3. No new primary dataset was generated.

## Supplementary Materials

The following supporting information can be downloaded at: https://www.mdpi.com/article/10.3390/nu18152483/s1, Supplementary File S1: PRISMA 2020 Checklist [5]. Supplementary File S2: Complete search strategies, retrieval/exclusion log, detailed extraction, outcome synthesis, and risk-of-bias assessment [25,26,27,28,29,30,31,32]. Supplementary File S3: Outcome-specific GRADE evidence profile and summary of findings.
