# Supplementary material for: DHA Alone, EPA + DHA, EPA + DHA + GLA, and EPA + DHA with or Without Vitamin D During Chemotherapy in Women with Breast Cancer: An Intervention- and Outcome-Stratified Systematic Review
Source: Nutrients. 2026 Aug 1;18(15):2483. doi: 10.3390/nu18152483 (PMC13468582; doi:10.3390/nu18152483)
Supplement: Supplementary file 1 [file nutrients-18-02483-s001.zip › Supplementary File S3.pdf]

Supplementary File S3

Outcome-specific GRADE evidence profile and summary of findings

Because no meta-analysis was performed, effect summaries are narrative. Certainty was judged by outcome domain using outcome-specific risk-of-bias judgments, inconsistency, indirectness, imprecision, and concern about missing results. K.F. performed the initial GRADE assessments, which were checked by M.W. and E.Ł.; disagreements were resolved by discussion. Publication bias was not formally tested because only ten heterogeneous trial clusters were available.

Outcome domain	Studies / participants	Narrative effect summary	Risk of bias	Inconsistency	Indirectness	Imprecision	Missing results / publication bias	Overall certainty	Interpretation
Safety/tolerability	10 trial clusters; denominators varied	No serious event directly attributed to supplementation; mild GI events in Almassri; adverse-event reporting variable.	Serious	Not serious	Not serious	Serious	Not formally assessed; concern cannot be excluded	Low	Small studies and non-standardized safety reporting limit confidence.
Inflammatory/immune/biological markers	Darwito; Arsic; DHA-WIN companions; Almassri companion; Mantha/DHALYA	Ki-67/VEGF, IL-6, cytokine/oxylipin/TIL, immune-cell, and compliance-restricted fatty-acid/protein-metabolism signals; heterogeneous and often exploratory or co-supplemented.	Serious	Serious	Serious	Serious	Not formally assessed; concern cannot be excluded	Very low	Biomarker effects do not establish patient-important clinical benefit.
CIPN / P-APS	Ghoreishi and Tawfik; 106 analyzed	Ghoreishi favorable for CIPN incidence; Tawfik neutral for P-APS/CIPN.	Serious	Serious	Not serious	Serious	Not formally assessed; concern cannot be excluded	Very low	Findings are inconsistent and imprecise.
Nutritional status / body composition	Almassri, de la Rosa Oliva, and Arsic; 169 analyzed/completed	Positive nutritional-status signal was confined mainly to the combined EPA+DHA+vitamin D arm; placebo-controlled body-composition findings in de la Rosa Oliva and Arsic were largely neutral.	Serious	Serious	Serious	Serious	Not formally assessed; concern cannot be excluded	Very low	Open-label co-supplementation limits attribution, while two placebo-controlled body-composition trials were neutral.
Functional/respiratory/lipid-lactate	Suzumura; 32 participants	Favorable respiratory muscle, 6-minute walk, lactate, and lipid measures.	Serious	Not estimable	Serious	Very serious	Not formally assessed; concern cannot be excluded	Very low	Single small no-placebo study; unreplicated outcome domain.
Quality of life / patient-reported outcomes	Almassri companion; DHA-WIN/Douglas; de la Rosa Oliva	Mixed: open-label factorial trial positive for selected outcomes; placebo-controlled DHA-WIN neutral.	Serious	Serious	Serious	Serious	Not formally assessed; concern cannot be excluded	Very low	Subjective outcomes, co-supplementation, and imprecision.
Anticancer response / survival	Bougnoux, Darwito, DHA-WIN, and Mantha/DHALYA; denominators varied	Bougnoux non-comparative response data; Darwito favorable HRs; DHA-WIN did not establish consistent benefit; DHALYA was terminated early and underpowered for survival.	Very serious	Serious	Serious	Serious	Not formally assessed; concern cannot be excluded	Very low	Favorable signals arise from non-comparative or single small trial evidence and are not consistent.

Downgrading explanations

Risk of bias: downgraded on the basis of outcome-specific RoB 2-informed judgments for attrition/per-protocol analyses, open-label no-placebo designs, non-comparative evidence, limited randomization reporting, and/or selective emphasis on multiple exploratory outcomes.

Inconsistency: downgraded when direction of effect differed between trials or when different interventions and outcome definitions precluded a coherent common effect.

Indirectness: downgraded when an active co-supplement was included, when evidence was limited to mechanistic biomarkers, or when a single-arm design did not directly estimate comparative efficacy.

Imprecision: downgraded because sample sizes were small, confidence intervals were wide or unavailable, and most domains were represented by one or two trial clusters.

Missing results/publication bias: formal testing was not performed. The domain was not used as an automatic downgrade in every row; however, concern cannot be excluded because the evidence base is small and heterogeneous.

Summary conclusion. Certainty is low for safety/tolerability and very low for all efficacy-related domains. The evidence does not support a clinical recommendation for DHA alone, EPA+DHA or fish oil without another active nutrient, EPA+DHA+GLA, or EPA+DHA with or without vitamin D during chemotherapy for breast cancer.
