# Supplementary material for: DHA Alone, EPA + DHA, EPA + DHA + GLA, and EPA + DHA with or Without Vitamin D During Chemotherapy in Women with Breast Cancer: An Intervention- and Outcome-Stratified Systematic Review
Source: Nutrients. 2026 Aug 1;18(15):2483. doi: 10.3390/nu18152483 (PMC13468582; doi:10.3390/nu18152483)
Supplement: Supplementary file 1 [file nutrients-18-02483-s001.zip › Supplementary File S2.pdf]

Supplementary File S2

Complete search strategies, retrieval/exclusion log, detailed extraction, outcome synthesis, and risk-of-bias assessment

Manuscript: DHA Alone, EPA+DHA, EPA+DHA+GLA, and EPA+DHA with or without Vitamin D During Chemotherapy in Women with Breast Cancer: An Intervention- and Outcome-Stratified Systematic Review

Companion reports from the same clinical trial were grouped at the trial level and were not double-counted. Randomized/enrolled and analyzed/completed denominators are reported separately.

S1. Information sources, search dates, and record counts

Source	Date searched	Search summary / limits	Records
PubMed/MEDLINE	8 July 2026	"omega-3" AND "breast cancer"; randomized clinical trial/clinical study; 1996-2026; humans/female where available	48
Cochrane Library/CENTRAL	8 July 2026	"omega-3" AND "breast cancer" AND "chemotherapy"; trial records screened for eligible prospective interventions	42
ClinicalTrials.gov	8 July 2026	Condition: breast cancer; other terms: omega-3 and chemotherapy; completed studies; female participants	7
Scopus	8 July 2026	Expanded TITLE-ABS-KEY strategy for breast cancer, omega-3/EPA/DHA, chemotherapy, and intervention/trial concepts	153
Web of Science Core Collection	8 July 2026	Expanded TS strategy for breast cancer, omega-3/EPA/DHA, chemotherapy, and intervention/trial concepts	174
Embase.com	8 July 2026	Expanded Emtree/free-text strategy for breast tumor, omega-3/EPA/DHA, chemotherapy, and intervention/trial concepts; humans	249
Citation/manual search	Completed 8 July 2026	Backward/forward citation searching of included reports and relevant reviews; trial registrations and companion reports checked	1 additional unique eligible report (Mantha et al.; NCT01548534)
Database/register total	-	Records identified in databases/registers before duplicate removal	673
Duplicates removed	-	DOI and normalized-title matching followed by manual verification	166
Records screened	-	Records after duplicate removal	507

S2. Full source-specific search strings

PubMed/MEDLINE (8 July 2026; n=48)

"omega-3" AND "breast cancer". Filters: randomized clinical trial OR clinical study; publication years 1996-2026; humans/female where available.

Cochrane Library/CENTRAL (8 July 2026; n=42)

"omega-3" AND "breast cancer" AND "chemotherapy". Trial records were screened for prospective interventions in women receiving chemotherapy for breast cancer.

ClinicalTrials.gov (8 July 2026; n=7)

Condition/disease: breast cancer; other terms: omega-3 AND chemotherapy; recruitment status: completed; sex: female.

Scopus (8 July 2026; n=153)

TITLE-ABS-KEY(("breast cancer" OR "breast neoplasm*" OR "breast carcinoma*" OR "mammary cancer*" OR (breast W/3 (cancer* OR neoplasm* OR carcinoma* OR tumor* OR tumour*))) AND ("omega-3" OR "omega 3" OR "n-3 PUFA*" OR "n 3 PUFA*" OR "fish oil*" OR "eicosapentaenoic acid*" OR "docosahexaenoic acid*" OR (EPA W/3 (omega* OR "n-3" OR fatty OR acid* OR supplement*)) OR (DHA W/3 (omega* OR "n-3" OR fatty OR acid* OR supplement*))) AND (chemotherap* OR neoadjuvant OR adjuvant OR paclitaxel OR docetaxel OR doxorubicin OR cyclophosphamide OR taxane*) AND (random* OR trial* OR placebo* OR intervention* OR supplement*))

Web of Science Core Collection (8 July 2026; n=174)

TS=(("breast cancer" OR "breast neoplasm*" OR "breast carcinoma*" OR "mammary cancer*" OR (breast NEAR/3 (cancer* OR neoplasm* OR carcinoma* OR tumor* OR tumour*))) AND ("omega-3" OR "omega 3" OR "n-3 PUFA*" OR "n 3 PUFA*" OR "fish oil*" OR "eicosapentaenoic acid*" OR "docosahexaenoic acid*" OR (EPA NEAR/3 (omega* OR "n-3" OR fatty OR acid* OR supplement*)) OR (DHA NEAR/3 (omega* OR "n-3" OR fatty OR acid* OR supplement*))) AND (chemotherap* OR neoadjuvant OR adjuvant OR paclitaxel OR docetaxel OR doxorubicin OR cyclophosphamide OR taxane*) AND (random* OR trial* OR placebo* OR intervention* OR supplement*))

Embase.com (8 July 2026; n=249)

('breast tumor'/exp OR 'breast cancer':ti,ab,kw OR 'breast neoplasm*':ti,ab,kw OR 'breast carcinoma*':ti,ab,kw OR 'mammary cancer*':ti,ab,kw OR ((breast OR mammary) NEAR/3 (cancer* OR neoplasm* OR carcinoma* OR tumor* OR tumour*)):ti,ab,kw) AND ('omega 3 fatty acid'/exp OR 'fish oil'/exp OR 'eicosapentaenoic acid'/exp OR 'docosahexaenoic acid'/exp OR 'omega-3':ti,ab,kw OR 'omega 3':ti,ab,kw OR 'n-3 PUFA*':ti,ab,kw OR 'fish oil*':ti,ab,kw OR 'eicosapentaenoic acid*':ti,ab,kw OR 'docosahexaenoic acid*':ti,ab,kw OR ((EPA OR DHA) NEAR/3 (omega* OR 'n-3' OR fatty OR acid* OR supplement*)):ti,ab,kw) AND ('chemotherapy'/exp OR 'cancer chemotherapy'/exp OR 'neoadjuvant therapy'/exp OR 'adjuvant chemotherapy'/exp OR chemotherap*:ti,ab,kw OR neoadjuvant:ti,ab,kw OR adjuvant:ti,ab,kw OR paclitaxel:ti,ab,kw OR docetaxel:ti,ab,kw OR doxorubicin:ti,ab,kw OR cyclophosphamide:ti,ab,kw OR taxane*:ti,ab,kw) AND (random*:ti,ab,kw OR trial*:ti,ab,kw OR placebo*:ti,ab,kw OR intervention*:ti,ab,kw OR supplement*:ti,ab,kw) AND [humans]/lim

Trial-identifier/citation audit (completed 8 July 2026; n=1 additional eligible report)

Backward and forward citations and trial registration identifiers were cross-checked. This identified the full peer-reviewed Mantha et al. DHALYA report (NCT01548534), which was not counted among the 673 database/register records.

S3. Full-text retrieval and exclusion log

Report / record	Final status	Reason	PRISMA category
Hashemipour MA, Barzegari S, Kakoie S, Habib Aghahi R. Effects of Omega-3 Fatty Acids Against Chemotherapy-induced Mucositis: A Double-blind Randomized Clinical Trial. Wounds. 2017;29(12):360-366.	Excluded	Mixed oncology population; breast-cancer subgroup data not separately extractable.	Wrong/mixed population
Anoushirvani AA, Poorsaadat L, Aghabozorgi R, Kasravi M. Comparison of the Effects of Omega 3 and Vitamin E on Paclitaxel-Induced Peripheral Neuropathy. Open Access Maced J Med Sci. 2018;6(10):1857-1861. doi:10.3889/oamjms.2018.333.	Excluded	Mixed cancer population (including breast, lung, and ovarian cancer); breast-cancer subgroup not separately extractable.	Wrong/mixed population
Mansara P, Deshpande RA, Vaidya MM, Kaul-Ghanekar R. Improved antioxidant status by omega-3 fatty acid supplementation in breast cancer patients undergoing chemotherapy: a case series. J Med Case Rep. 2015;9:148. doi:10.1186/s13256-015-0619-3.	Excluded	Case series of five patients; ineligible design.	Ineligible design

Report / record	Final status	Reason	PRISMA category
Arcos D, Ng DQ, Ke Y, Toh YL, Chan A. Prediction of gastrointestinal symptoms trajectories using omega-3 and inflammatory biomarkers in early-stage breast cancer patients receiving chemotherapy. Support Care Cancer. 2024;32:76. doi:10.1007/s00520-023-08274-5.	Excluded	Observational/biomarker analysis; no assigned EPA/DHA supplementation effect.	No supplementation intervention effect
Newell M, Mackey JR, Bigras G, et al. Comparing DHA concomitant with neoadjuvant chemotherapy versus neoadjuvant chemotherapy alone in breast cancer (DHA-WIN): protocol. BMJ Open. 2019;9:e030502. doi:10.1136/bmjopen-2019-030502.	Excluded	Protocol only; no outcome data. Outcome reports from the same trial were included.	Protocol without outcomes
ClinicalTrials.gov NCT01049295 / preliminary registry record corresponding to Ghoreishi et al.	Excluded	Registry/preliminary record corresponding to an included full report; no independent outcome report.	Registry without independent outcomes
de la Rosa Oliva et al. SABCS conference abstract, 2017.	Excluded	Preliminary conference abstract duplicating the included 2019 full report.	Duplicate preliminary report
Bougnoux P, et al. DHALYA phase III conference abstract. Cancer Res. 2015;75(9 Suppl):Abstract P3-13-08; NCT01548534.	Excluded	Preliminary conference abstract linked to the included full peer-reviewed Mantha et al. report; insufficient for independent extraction and not counted as a separate report.	Duplicate preliminary report / linked abstract

All 21 reports from database/register screening and the one additional report identified through trial-identifier/citation searching were obtained and assessed; none was unavailable. The DHALYA conference abstract was linked to the full peer-reviewed Mantha et al. report and was not counted separately. The Suzumura report was evaluated from the retrieved full-text article; information absent from the article is explicitly recorded as not reported in the full text.

S4A. Trial-level characteristics and intervention details

ID	Trial cluster	Country	Design	Sample	Population / chemotherapy	Intervention	Comparator	Duration	Registration	Funding / COI
S1	Ghoreishi 2012	Iran	Double-blind placebo RCT	69 randomized; 57 analyzed	Breast cancer; paclitaxel	DHA-predominant EPA+DHA product, 640 mg TID; 54% DHA, 10% EPA; author-reported total omega-3 1244.1 mg/day	Sunflower-oil placebo	During paclitaxel + 1 month	NCT01049295	No formal funding statement was reported. Omega-3 pearls were supplied by Minami Nutrition and placebo pearls by Dana Pharma; authors declared no competing interests.
S2	Bougnoux 2009	France	Open-label single-arm phase II	25 enrolled/analyzed	Visceral metastatic; first-line FEC	DHA 1.8 g/day; 7-10 day loading then approximately 5 months	None	16-22 weeks	NR	Supported by grants from the French Ministry of Health (PHRC), INSERM, Tours University Hospital, University François Rabelais, and the Indre-et-Loire section of the French League Against Cancer; DHASCO capsules were provided by Martek Biosciences; authors declared no conflict of interest.
S3	Darwito 2019	Indonesia	Double-blind RCT	48 randomized/analyzed (24/24 reported)	Stage IIIB; neoadjuvant CAF x3 then mastectomy	Fish-oil omega-3 1 g/day	Placebo	51 days	NR	Partially supported by extramural funding from the Faculty of Medicine, Universitas Diponegoro, and Universitas Gadjah Mada; funders had no influence on study design or manuscript preparation; authors

ID	Trial cluster	Country	Design	Sample	Population / chemotherapy	Intervention	Comparator	Duration	Registration	Funding / COI
										declared no financial or professional competing interests.
S4	Tawfik 2023	United States	Pilot double-blind placebo RCT	60 randomized; 49 evaluable	Breast cancer; weekly paclitaxel	EPA+DHA ethyl esters (Lovaza) 4 g/day	Placebo	1 week before through paclitaxel	NCT01821833	Supported by the National Cancer Institute under awards UG1CA189823 and UG1CA232760, with partial support from NCI P30CA118100 and the Biostatistics shared resource; authors declared no competing interests.
S5	Arsic 2023	Serbia	Double-blind placebo RCT	32 randomized; 29 completed	Postmenopausal stage IIA-IIIA; adjuvant anthracycline chemotherapy	EPA 400 mg + DHA 600 mg + GLA 351 mg/day	Mineral-oil placebo	12 weeks	NCT03516253	Supported by the Ministry of Education, Science and Technological Development and Innovation of the Republic of Serbia (contract 451-03-47/2023-01/200015); authors declared no competing interests.
S6	Almassri 2024/2025	Palestine	Open-label 1:1:1:1 randomized factorial trial	96 recruited/randomized; 88 analyzed (22/arm)	Stage II-III; AC chemotherapy	EPA+DHA: EPA 360 mg + DHA 240 mg/day; vitamin D3 50,000 IU/week; combined EPA+DHA+vitamin D arm	No-supplementation control and factorial arms	9 weeks	NCT05331807	2024 report: no external funding; self-funded by the first author; authors declared no conflict of interest. 2025 companion report: no specific grant from public, commercial, or not-for-profit funding agencies; authors declared no competing interests.
S7	DHA-WIN 2025	Canada	Phase II double-blind placebo RCT	76 randomized; 49 completed	Non-metastatic; six cycles neoadjuvant chemotherapy	Algae-derived DHA 4.4 g/day	Corn/soy-oil placebo	18 weeks	NCT03831178	Supported by the Canadian Institutes of Health Research (RES0037745), Cross Cancer Institute Investigator Initiated Trials (IIT-0005), and a gift from the Butler Family Foundation; authors declared no competing interests.
S8	de la Rosa Oliva 2019	Mexico	Double-blind placebo RCT	53 randomized; 52 analyzed	Locally advanced; AC then paclitaxel +/- trastuzumab	EPA 1.6 g + DHA 0.8 g/day	Sunflower-oil placebo	6 months	NR	No funding or conflict-of-interest statement was reported in the full text.
S9	Suzumura 2016	Brazil	Randomized no-supplementation controlled study	32 randomized/analyzed (15 control; 17 fish oil)	Women at beginning of chemotherapy	Fish oil 4 g/day; approximately EPA 0.8 g + DHA 0.4 g/day	No supplementation	60 days	NR	Fish-oil capsules were donated by Naturalis; no other funding or conflict-of-interest statement was reported in the full text.
S10	Mantha/DHALYA 2022	France	Randomized double-blind matched-control phase III trial; terminated early	63 randomized (32 control/31 fish oil); 46 completed	Metastatic HER2-negative, HR-positive breast cancer; planned first-line chemotherapy	Three 200 mL cans/day; EPA 2.64 g + DHA 1.56 g/day	Isocaloric/macronutrient-matched control supplement with copra oil	Approximately 10-day pre-chemotherapy loading; 3 months total	NCT01548534	Funded by PHRC (11-153), University of Tours, Région Centre-Val de Loire, INSERM, Canceropôle Grand Ouest, Ligue Nationale Contre le Cancer, CANCEN, ACORT, Inserm Cancer, and INCa (PLBIO14-213); authors declared no conflict of interest.

S4B. Report-level outcomes and numerical results

Report / study	Citation	Outcome / time point	Numerical result or report-level finding	Interpretation	Safety / registration
R1 / S1	Ghoreishi 2012	CIPN; end of treatment/follow-up	CIPN occurred in 9/30 (30.0%) vs 16/27 (59.3%); OR for CIPN incidence 0.30, 95% CI 0.10-0.88, p=0.029; severity p=0.054; author-reported NNT=3.	Favorable incidence signal, but 12/69 randomized participants were excluded from analysis.	Trial registered; supplementation-related serious AEs not reported.
R2 / S2	Bougnoux 2009	Response, TTP, OS, safety	ORR 44% (95% CI 24.5-63.5); median TTP 6 months; median OS 22 months; grade 3/4 neutropenia 80%.	Non-comparative hypothesis-generating evidence; subgroup by plasma incorporation is post-baseline.	No DHA-related adverse events reported.
R3 / S3	Darwito 2019	Ki-67, VEGF, OS, DFS	Greater Ki-67 reduction p=0.032; VEGF reduction p=0.041; OS HR 0.411 (95% CI 0.201-0.840); DFS HR 0.439 (95% CI 0.222-0.869).	Statistically favorable but single small trial with multiple outcomes and limited reporting.	AEs/registration NR.
R4 / S4	Tawfik 2023	P-APS and CIPN	Week-1 P-APS 17/25 (68.0%) vs 15/24 (62.5%), p=0.77; 12-week P-APS 21/25 (84.0%) vs 21/24 (87.5%), p=1.0; CIPN 12.8 vs 8.4, p=0.21.	Neutral/negative; pilot underpowered.	NCT01821833.
R5 / S5	Arsic 2023	IL-6, IL-8, IL-10, TNF-alpha, fatty-acid profile, body composition	Adjusted end-of-study IL-6 lower with EPA+DHA+GLA (p<0.01); no between-group difference in IL-8, IL-10, or TNF-alpha; no body-composition change.	The effect of EPA+DHA cannot be isolated from GLA.	29/32 completed; NCT03516253.
R6 / S6	Almassri 2024	PG-SGA, anthropometry, albumin, dietary intake, AEs	Combined EPA+DHA+vitamin D vs control: PG-SGA p<0.001, BMI p=0.032; albumin improved within combined group p=0.042 but between-group p=0.198; four mild AEs/88.	Positive nutritional signal driven by combined arm; open-label and no placebo.	NCT05331807.
R7 / S6	Almassri 2025 companion	Quality of life and inflammatory markers	Selected QoL and inflammatory-marker improvements reported across the EPA+DHA-only, vitamin-D-only, and combined EPA+DHA+vitamin D arms; report-specific estimates retained in source tables.	Factorial/open-label design prevents attribution to EPA+DHA alone.	Same NCT05331807 trial; do not double-count sample.
R8 / S7	DHA-WIN primary 2025	Safety, Ki-67, pathological response, longer-term clinical outcomes	Primary randomized report did not establish a consistent improvement in pathological response, Ki-67, or clinical efficacy with DHA.	Negative/uncertain clinical efficacy; high attrition (49/76 completed).	NCT03831178; generally tolerated.
R9 / S7	DHA-WIN cytokine/oxylipin/TIL companion 2025	Plasma cytokines, oxylipins, tumor-infiltrating lymphocytes	Multiple mechanistic changes were explored; no single prespecified clinical effect estimate and no consistent clinical translation.	Exploratory multiplicity and companion-report context.	Same trial cluster.
R10 / S7	DHA-WIN PBMC companion 2025	Peripheral blood immune cells and function	NLR maintained with DHA but increased with placebo (interaction p=0.02); selected T-cell/monocyte measures were attenuated.	Mechanistic secondary outcomes; no established patient benefit.	Same trial cluster; NCT03831178.
R11 / S7	Douglas/DHA-WIN QoL companion 2025	QoL and exercise behavior	No significant adjusted between-group change in any QoL indicator; aerobic exercise interaction p=0.53; resistance training interaction p=0.15.	Neutral patient-reported/behavioral outcome.	49 trial completers; same trial cluster.
R12 / S8	de la Rosa Oliva 2019	Toxicity, symptoms, body composition, cardiometabolic profile, QoL	No significant differences in most outcomes; xerostomia improved between groups p=0.032.	Isolated symptom signal among multiple neutral outcomes.	52/53 analyzed.

Report / study	Citation	Outcome / time point	Numerical result or report-level finding	Interpretation	Safety / registration
R13 / S9	Suzumura 2016	Respiratory strength/endurance, 6-minute walk, lactate, lipids	Improved maximal inspiratory/expiratory pressure, maximum voluntary ventilation, 6-minute walk, lactate, and selected lipid measures (reported $p<=0.05$).	Single small no-placebo study; hypothesis-generating.	Adverse events and trial registration were not reported in the full text.
R14 / S10	Mantha/DHALYA 2022	Plasma fatty acids, proinflammatory cytokines, and natural isotopic markers; baseline, approximately 10 days, and 3 months	Plasma EPA/DHA increased rapidly with fish oil; compliance-restricted analyses suggested an early protein-metabolism change, while proinflammatory cytokines decreased only after longer supplementation.	Mechanistic signal; early termination, declining compliance, small exploratory isotopic subsample, and no adequately powered survival analysis.	NCT01548534; dedicated comparative adverse-event results were not reported in this mechanistic publication.

S5. Outcome-level narrative synthesis

Outcome domain	Contributing evidence	Direction of evidence	Interpretation
Inflammatory/immune/biological markers	Darwito; Arsic; DHA-WIN companions; Almassri companion; Mantha/DHALYA	Several biomarker/immune signals, including a compliance-restricted metabolic/cytokine signal, but findings were heterogeneous, often exploratory, and sometimes co-supplemented.	Biologically plausible but very low certainty; do not infer clinical efficacy.
CIPN / P-APS	Ghoreishi; Tawfik	One favorable incidence trial and one neutral pilot trial.	Inconsistent and very low certainty.
Nutritional status / body composition	Almassri; de la Rosa Oliva; Arsic	Positive signal from combined EPA+DHA+vitamin D; body-composition findings in de la Rosa Oliva and Arsic were neutral.	Potential signal limited by co-supplementation and risk of bias.
Functional/respiratory/lipid-lactate	Suzumura	Favorable respiratory, walking, lactate, and lipid outcomes.	Single small no-placebo trial; very low certainty.
Quality of life / PROs	Almassri companion; DHA-WIN/Douglas; de la Rosa Oliva	Mixed; open-label co-supplementation positive, DHA-WIN neutral.	No firm QoL conclusion.
Anticancer response / survival	Bougnoux; Darwito; DHA-WIN; Mantha/DHALYA	Favorable non-comparative/small-trial signals were not reproduced in a consistent randomized evidence base; DHALYA was terminated early and underpowered for survival.	Insufficient evidence for treatment enhancement.
Safety / tolerability	All 10 trial clusters	No serious supplementation-related adverse event directly attributed; reporting varied.	Directionally reassuring but low certainty.

S6A. Outcome-specific RoB 2-informed domain judgments for randomized trials

Judgments are outcome-specific and use only the standard RoB 2 categories: low risk, some concerns, and high risk. Separate rows are shown when a trial contributed outcome sets with materially different bias considerations.

Study / assessed result(s)	Randomization process	Deviations from intended interventions	Missing outcome data	Outcome measurement	Selection of reported result	Overall risk	Rationale
Ghoreishi 2012 - CIPN incidence	Some concerns	Low risk	High risk	Low risk	Some concerns	High risk	69 randomized and 57 analyzed; attrition and per-protocol analysis materially affect the CIPN result.
Darwito 2019 - Ki-67/VEGF and survival	Some concerns	Some concerns	Low risk	Low risk	High risk	High risk	Randomization and blinding details were limited; multiple biomarker and survival outcomes raise concern about selection of the reported result.
Tawfik 2023 - P-APS/CIPN	Some concerns	Low risk	Some concerns	Low risk	Some concerns	Some concerns	Pilot trial with 49 of 60 participants evaluable; outcomes were prespecified, but missing data and imprecision remain concerns.
Arsic 2023 - inflammatory biomarkers/body composition	Some concerns	Low risk	Some concerns	Low risk	Some concerns	Some concerns	Twenty-nine of 32 participants completed follow-up; placebo controlled. GLA co-administration affects GRADE indirectness rather than internal risk of bias.
Almassri 2024 - nutritional/anthropometric outcomes	Some concerns	High risk	Some concerns	Some concerns	Some concerns	High risk	Open-label, no-placebo factorial trial; 88 of 96 participants analyzed. Knowledge of allocation could influence adherence and PG-SGA-related assessments.
Almassri 2025 companion - QoL/inflammatory outcomes	Some concerns	High risk	Some concerns	High risk	Some concerns	High risk	Open-label, no-placebo design creates substantial concern for subjective QoL measurement and deviations from intended interventions.
DHA-WIN primary 2025 - clinical/pathological outcomes	Low risk	Low risk	High risk	Low risk	Some concerns	High risk	Only 49 of 76 randomized participants completed the intervention, creating high risk from missing outcome data.
DHA-WIN companions 2025 - mechanistic/QoL outcomes	Low risk	Low risk	High risk	Low risk	High risk	High risk	Substantial attrition and exploratory companion analyses with multiple outcomes create high risk from missing data and selection of reported results.
de la Rosa Oliva 2019 - toxicity/body composition/QoL	Some concerns	Low risk	Low risk	Low risk	Some concerns	Some concerns	Fifty-two of 53 participants were analyzed; randomization details and multiple reported outcomes leave some concerns.
Suzumura 2016 - functional/lipid outcomes	Some concerns	High risk	Low risk	Low risk	Some concerns	High risk	No placebo or blinding; all 32 randomized participants were analyzed. Objective measurements reduce, but do not eliminate, outcome-measurement concerns.

Study / assessed result(s)	Randomization process	Deviations from intended interventions	Missing outcome data	Outcome measurement	Selection of reported result	Overall risk	Rationale
Mantha/DHALYA 2022 - inflammatory/metabolic outcomes	Low risk	Some concerns	High risk	Low risk	High risk	High risk	Web-based stratified randomization and double blinding were reported, but the trial ended early; 46/63 completed, principal analyses were restricted to compliant subsets, and multiple exploratory mechanistic outcomes were reported.

S6B. Appraisal of the non-comparative phase II study

Study	Prospective design	Completeness	Principal limitations	Overall interpretation
Bougnoux 2009	Prospective enrollment and clearly described intervention/outcomes	25 participants; follow-up reported	No concurrent comparator; subgroup by DHA incorporation was post-baseline	High concern for causal/comparative efficacy; suitable only for hypothesis generation

S7. Record management and companion-report rules

Deduplication: exports were merged and duplicates were identified using exact DOI matching and normalized-title matching (case/punctuation/whitespace normalization), followed by manual verification of possible matches. A total of 166 duplicate records were removed.

Companion reports: the Almassri 2024 and 2025 papers were treated as two reports from one factorial trial. The four DHA-WIN publications were treated as reports from one randomized trial. The DHALYA conference abstract was linked to the full Mantha et al. report and was not counted as a separate report. Participant denominators were counted only at the trial-cluster level and are displayed as randomized/enrolled and analyzed/completed rather than combined into one cross-study total. The final evidence base comprised 14 reports from 10 trial clusters.

Abbreviations: AC, doxorubicin/cyclophosphamide; AEs, adverse events; CAF, cyclophosphamide/doxorubicin/5-fluorouracil; CI, confidence interval; CIPN, chemotherapy-induced peripheral neuropathy; DFS, disease-free survival; DHA, docosahexaenoic acid; EPA, eicosapentaenoic acid; FEC, 5-fluorouracil/epirubicin/cyclophosphamide; GLA, gamma-linolenic acid; HER2, human epidermal growth factor receptor 2; HR, hormone receptor; NLR, neutrophil-to-lymphocyte ratio; NNT, number needed to treat; NR, not reported; ORR, objective response rate; OS, overall survival; P-APS, paclitaxel-associated acute pain syndrome; PG-SGA, Patient-Generated Subjective Global Assessment; PRO, patient-reported outcome; PUFA, polyunsaturated fatty acid; QoL, quality of life; RCT, randomized controlled trial; TTP, time to progression.
